# Reciprocal regulation of A-to-I RNA editing and the vertebrate nervous system

**DOI:** 10.3389/fnins.2013.00061

**Published:** 2013-04-18

**Authors:** Andrew C. Penn, Ales Balik, Ingo H. Greger

**Affiliations:** ^1^Interdisciplinary Institute for Neuroscience, Université de Bordeaux, UMR 5297Bordeaux, France; ^2^CNRS, Interdisciplinary Institute for Neuroscience, UMR 5297Bordeaux, France; ^3^Department of Neurophysiology, Institute of Physiology, Academy of Sciences of the Czech Republic v.v.i.Prague, Czech Republic; ^4^Neurobiology Division, MRC Laboratory of Molecular BiologyCambridge, UK

**Keywords:** AMPA receptor, A-to-I RNA editing, dynamics of RNA editing, R/G editing site, ADAR2

## Abstract

The fine control of molecules mediating communication in the nervous system is key to adjusting neuronal signaling during development and in maintaining the stability of established networks in the face of altered sensory input. To prevent the culmination of pathological recurrent network excitation or debilitating periods of quiescence, adaptive alterations occur in the signaling molecules and ion channels that control membrane excitability and synaptic transmission. However, rather than encoding (and thus “hardwiring”) modified gene copies, the nervous systems of metazoa have opted for expanding on post-transcriptional pre-mRNA splicing by altering key encoded amino acids using a conserved mechanism of A-to-I RNA editing: the enzymatic deamination of adenosine to inosine. Inosine exhibits similar base-pairing properties to guanosine with respect to tRNA codon recognition, replication by polymerases, and RNA secondary structure (i.e.,: forming-capacity). In addition to recoding within the open reading frame, adenosine deamination also occurs with high frequency throughout the non-coding transcriptome, where it affects multiple aspects of RNA metabolism and gene expression. Here, we describe the recoding function of key RNA editing targets in the mammalian central nervous system and their potential to be regulated. We will then discuss how interactions of A-to-I editing with gene expression and alternative splicing could play a wider role in regulating the neuronal transcriptome. Finally, we will highlight the increasing complexity of this multifaceted control hub by summarizing new findings from high-throughput studies.

## A-to-I RNA editing in the vertebrate nervous system

Although progress has been made in characterizing the functions of invertebrate editing sites, the challenge of understanding the true scale and roles of RNA editing in regulating neurophysiology in higher vertebrates continues at a somewhat slower pace. In particular, the impact of editing in non-coding regions, which harbor the vast majority of editing sites (see below) is not known. Base changes via RNA editing expand on the central dogma of molecular biology by readjusting the genetic code at the RNA level in order to substitute amino acids (Rosenthal and Seeburg, 2012). Remarkably, this occurs at functionally critical positions in targets mediating synaptic transmission. For example, editing of the α3 subunits of GABA_A_ receptor ion channels modulates agonist potency and receptor gating properties to tune inhibition (Ohlson et al., 2007; Rula et al., 2008). Similarly, at excitatory synapses A-to-I editing is responsible for a number of recoding events in many of the non-NMDA glutamate receptor ion channel subunits (AMPA GluA2, 3, 4, and kainate GluK1, 2; Sommer et al., 1991; Köhler et al., 1993; Lomeli et al., 1994), including the efficient Q/R site conversion of GluA2, which gates calcium permeability and receptor trafficking that are essential for survival (Sommer et al., 1991; Burnashev et al., 1992; Brusa et al., 1995; Greger et al., 2002). More generally, membrane excitability is modified by A-to-I editing of select subunits of voltage-gated potassium (Kv1.1) and calcium (Cav1.3) channels resulting in altered channel inactivation properties (Bhalla et al., 2004; Huang et al., 2012). Neuromodulatory control by serotonin is also targeted, where A-to-I editing of the metabotropic receptor 5-HT_2C_ attenuates coupling to its G-protein second messenger system (Burns et al., 1997). Furthermore, editing could also regulate serotonin signaling more globally by modifying activity of the enzyme tryptophan hydroxylase-2 (TPH2, Grohmann et al., 2010), which is rate-limiting for serotonin synthesis in the brain (Zhang et al., 2004). As the list of non-synonymous codon- changes in neuron-related transcripts continues to grow (e.g., Danecek et al., 2012), it appears that A-to-I RNA editing is poised to directly tune the function of key nervous system components. This is particularly evident in invertebrates where recoding sites are more frequent and where functional changes have been elucidated (e.g., Rosenthal and Bezanilla, 2002; Hoopengardner et al., 2003; Colina et al., 2010). In fact, a recent study showed that editing of delayed rectifier potassium channels mediates temperature adaptation (Garrett and Rosenthal, 2012) to compensate for an overall slower signaling at low temperatures, editing accelerates gating kinetics of this potassium channel in Arctic and Antarctic squid species relative to their tropical relatives (Garrett and Rosenthal, 2012). Invertebrate RNA editing is beyond the scope of our discussion and so we refer the reader to some recent reviews (Jepson and Reenan, 2008; Rieder and Reenan, 2012).

## Developmental regulation of A-to-I editing

The deamination reactions responsible for A-to-I editing are catalyzed by a family of “editases”: adenosine deaminases acting on RNA (ADARs). The relatively high inosine content of brain mRNA (Paul and Bass, 1998), seizure susceptibility and lethal neurological phenotype of ADAR2 knockout mice (Higuchi et al., 2000), and the overall more selective expression of ADARs in the nervous system suggest that A-to-I editing contributes to refining neuronal function in development and during adult forms of synaptic plasticity. Developmental elevation of editing at various sites for many coding targets has been shown recently using new high-throughput sequencing technologies (Wahlstedt et al., 2009). These findings concur with earlier, more detailed studies on specific sites (e.g., Bernard and Khrestchatisky, 1994; Lomeli et al., 1994; Rula et al., 2008; Huang et al., 2012; Irimia et al., 2012). Age-dependent increases have also been documented for editing of small, non-coding RNA sequences, such as microRNAs (miRNAs), which typically bind to 3′ untranslated regions (UTRs) of transcripts to signal their degradation (Ekdahl et al., 2012). The potential for cross-talk between editing and gene expression control mechanisms in regulating neuronal development is exemplified with the case-study of miRNA cluster 379–410 (Ekdahl et al., 2012; Vesely et al., 2012). Here, editing in the critical seed regions of miRNA-381 and 376b prevents binding to Pumilio 2 (Pum2) mRNA, which codes for a translational repressor serving to negatively regulate outgrowth of neuronal dendrites. Consistent with this, developmental changes in editing of these miRNAs correlated with increased expression of Pum2 (Ekdahl et al., 2012). The increasing discovery of edits in non-coding sequences and the enrichment of some ADARs in the nervous system make it tempting to postulate that some observed tissue-specific expression patterns could result from editing-dependent switches in miRNA seed regions (Kawahara et al., 2007) or 3′-UTRs (Borchert et al., 2009), or from ADAR-modulated processing of microRNAs (Yang et al., 2006; Heale et al., 2009). Indeed, transcription profiling of the brain of ADAR2 knockout mice indicates editing could regulate the expression of a large number of genes (Horsch et al., 2011). Intriguingly, the genetic impact of A-to-I editing may be underestimated from mouse models since a disproportionately large amount of editing in humans also occurs in embedded primate-specific Alu elements that likely function to regulate gene expression (Maas, 2010).

## Cross-talk between A-to-I editing and alternative splicing

In addition to interactions with gene-expression control mechanisms, cross-talk exists between A-to-I editing and alternative splicing. Developmentally regulated, evolutionarily conserved RNA editing of transcripts encoding the central nervous system (CNS)-specific alternative splicing factor Nova1, reduces its degradation by the proteasome thereby increasing Nova1 protein levels (Irimia et al., 2012). Nova1 is expressed most in the ventral spinal cord where it is essential for normal postnatal motor function and notably regulates alternative splicing of multiple inhibitory synaptic targets, including the major scaffold protein gephyrin and the γ2 and α2 subunits of the GABA_A_ and glycine receptor ion channels, respectively (Jensen et al., 2000; Ule et al., 2005). It remains to be determined how changes in editing of endogenous Nova1 impact on the splicing of its targets, and whether or not aberrant Nova1 editing could aggravate motor neuron demise in sporadic amyotrophic lateral sclerosis (ALS); a condition strongly associated with deficient ADAR2 expression and GluA2 Q/R site editing (e.g., Hideyama et al., 2010, 2012). Feedback regulation of editing exists and occurs directly via alternative splicing: ADAR2 regulates splicing of its own pre-mRNA by creating a new splice acceptor site via its A-to-I editing activity. This causes an insertion of 47 nucleotides into the coding sequence and a frameshift resulting in a truncated, catalytically inactive protein (Rueter et al., 1999; Slavov and Gardiner, 2002; Feng et al., 2006). Another interesting example demonstrating the interaction of A-to-I editing with other RNA processes occurs in the 5-HT_2C_ receptor pre-mRNA. Here, an alternative splice donor site (necessary for the coding of a full-length receptor isoform) is silenced by a sequence element, which is weakened either by RNA editing (Flomen et al., 2004) or by an editing-independent mechanism that involves base-pairing of a small nucleolar RNA (snoRNA) HBII-52 (Kishore and Stamm, 2006). Consequently, neurons employ an unusual mechanism to regulate the editing of full-length 5-HT_2C_ receptors, which is significant in maintaining a normal serotonergic system and its associated impact on cognition and behavior (Kishore and Stamm, 2006; Doe et al., 2009; Morabito et al., 2010). Editing-dependent changes in splicing efficiency are also pivotal for AMPA-type glutamate receptor subunits: the essential Q/R recoding event in the GluA2 subunit, which controls ion channel calcium permeability, is associated with more efficient pre-mRNA splicing (Brusa et al., 1995). As a result, coupled editing and splicing ensures a significantly high fraction of Q/R-edited GluA2 mRNA to tolerate modest changes in ADAR2 activity (Schoft et al., 2007; Hideyama et al., 2012; Penn et al., 2013). Also in GluA2, a correlation between R/G site editing and alternative splice site selection appears to reflect a coupling associated with the homeostatic control of AMPA receptor biogenesis and function selectively in the CA1 region of the hippocampus (Penn et al., 2012; Balik et al., 2013).

## Neuronal activity driven regulation of RNA editing

The prospect of activity-dependent changes in A-to-I editing is an exciting recent development. There are various studies describing changes in A-to-I editing in diseases including ALS, epilepsy, and cancer, which mostly involve the GluA2 Q/R site and are associated with Ca^2+^ influx through AMPA receptors (Krestel et al., 2004; Maas et al., 2006). Another target is the serotonin receptor; of which altered G-protein coupling efficiencies of the 5-HT_2C_ receptor have been implicated in neuropsychiatric disorders (e.g., Gurevich et al., 2002; Bhansali et al., 2007; O'Neil and Emeson, 2012). However, one feature underlying many of these findings is that the pathological insults tend to have a dramatic impact on neuronal activity (e.g., stress, kindling, ischemia,). Some evidence points to a control of editing fundamentally by neuronal signaling. Early reports showed that serotonergic signaling via 5-HT_2C_ receptor could regulate editing of its own transcript to feedback onto the strength of receptor G-protein coupling (Gurevich et al., 2002). The same group later showed that the effect of a serotonin-selective reuptake inhibitor could reverse stress-induced changes in 5-HT_2C_ editing (Englander et al., 2005). Recent work in neuronal cultures derived from cerebral cortex has demonstrated that pharmacologically induced changes in neuronal activity can impact on ADAR targets (Orlandi et al., 2011; Sanjana et al., 2012). Altering neuronal activity in cultured hippocampal slices revealed analogous results, which turned out to be cell-type specific: editing changes occurred in the CA1, but not in CA3 subfield, which are composed of functionally and anatomically diverse neuronal cell types (Balik et al., 2013). Therefore, A-to-I editing has the capacity to fine-tune signaling in select neuronal circuitries. In two independent studies, chronic treatments lead to similar changes in AMPA receptor R/G site editing and concurrent changes in ADAR2 expression levels (Sanjana et al., 2012; Balik et al., 2013), which was also accompanied by regulation of ADAR2 self-editing (Balik et al., 2013). Interestingly, a recent study used a reporter based on the R/G site substrate to screen for repressors of ADAR2-mediated editing and identified three RNA-binding proteins (Tariq et al., 2013). The expression of two of these candidates, the splicing factor SFRS9 and the RNA helicase DDX15, was found to be regulated during mouse development and also responded to activity manipulations in CA1 of cultured hippocampal slices (Tariq et al., 2013). Binding of these factors around the R/G site might inhibit editing either by competing with ADAR2 for the substrate and/or by interacting directly with editase to reduce its activity (Tariq et al., 2013). A characterization of the physiological impact of editing site regulation in the plasticity of neuronal functions as well as an elucidation of cell-type/state specific changes in editing is now crucial and a very exciting prospect.

## Mechanisms underlying ADAR regulation

The mechanisms underlying editing regulation are currently unclear. These partly involve changes in ADAR levels (Balik et al., 2013), which, in the case of ADAR2, are under negative feedback control (Feng et al., 2006). However, this will depend on the efficiency of editing for a given ADAR substrate and is less likely to be relevant for strongly edited sites (Balik et al., 2013), such as the GluA2 Q/R site, for example. The “strength” of editing varies during development (Lomeli et al., 1994; Wahlstedt et al., 2009) and may be regulated in a cell- or tissue-selective manner. High-throughput sequencing data from cell lines imply overall low levels of editing (e.g., Bahn et al., 2012), but how this relates to editing levels in tissue remains to be established. Earlier reports described changes in ADAR expression levels during development (e.g., Paupard et al., 2000; Hang et al., 2008), but have recently been challenged as being responsible for observed editing site changes (Jacobs et al., 2009; Wahlstedt et al., 2009). Over the last decade, a great deal of emphasis has been placed on identifying and characterizing ADAR isoforms arising from alternatively spliced exons and transcription start sites (George et al., 2011). The varying activity of different ADAR isoforms has been described for some editing sites and so has their regulated expression during brain development (George et al., 2011), and the control of ADAR1 transcript levels by microRNAs (miRNA-1, Lim et al., 2005). The enigmatic, brain-specific (but non-catalytic) ADAR3 protein has been proposed to act in a dominant negative fashion on targets of other ADARs *in vitro* (Chen et al., 2000), but still little is known about its role and significance (Nishikura, 2010). More recently, protein structural studies have revealed candidates for the modulation of ADAR protein function. The ADAR2 catalytic domain contains a structurally integral inositol hexakisphosphate (IP_6_) required for efficient editing activity (MacBeth et al., 2005). An intriguing postulation is that elevated IP_6_ formed from phospholipase C (PLC) following 5-HT_2C_ activation might increase activity of nascent ADAR2 protein and account for some of the feedback onto 5-HT_2C_ receptor editing (Schmauss et al., 2010). However, further work is required to determine whether or not levels of IP_6_ in neurons are rate-limiting for ADAR2 activity. Post-translational modifications have also been shown to regulate ADAR protein function or abundance, including SUMOylation, phosphorylation-dependent propyl-isomerization and ubiquitination (Desterro et al., 2005; Marcucci et al., 2011). Furthermore, the control of dynamic associations of ADARs with subcellular compartments has been proposed as a means to sequester functional ADARs away from their targets in the nucleus. For example, induced translocation of ADAR2 (and likely also the short p110 form of ADAR1) from the nucleolus can increase activity at editing sites (Desterro et al., 2003; Sansam et al., 2003). However, contextual examples for this type of regulation in the nervous system remain elusive. Another example is the cytoplasmically localized p150 form of ADAR1, which is transcribed from an interferon-inducible promoter and can undergo regulated expression in some tissues, although not in the brain (Shtrichman et al., 2002; George et al., 2005). Further clues from pathology may reveal more candidate mechanisms relevant to physiological ADAR control. One example is the potential ADAR2 regulation by CA1-specific changes of cAMP-response element-binding protein (CREB) activity that occur following transient ischemic insults (Peng et al., 2006; Kitagawa, 2007). Consistent with these suggestions, the ADAR2 promoter contains a CREB/AP-1 binding site, which incidentally has shown necessary for ADAR2 regulation in glucose-responsive pancreatic cells via the stress-activated protein kinase JNK1 pathway (Yang et al., 2012). Furthermore, a link between calcium signaling via L-type voltage-gated calcium channels and activation of nuclear CREB might be key to understanding activity-dependent changes in ADAR2 expression (Wheeler et al., 2008; Balik et al., 2013). Challenges lie ahead to identify and detail the potential routes of ADAR regulation that are physiologically most relevant in different nervous system contexts.

## High-throughput sequencing and editing in non-coding regions

The lack of a clear signature for potential editing sites in gene sequence was limiting for the identification of new RNA editing sites (Hoopengardner et al., 2003). Before high-throughput sequencing techniques were available, a systematic search for new sites was based on computational analysis of the available databases containing genomic and transcriptional data. For example, human expressed sequence tags (ESTs) and cDNA data were aligned to genomic sequences to yield the discovery of four new genes subjected to editing (Clutterbuck et al., 2005; Levanon et al., 2005). However, these approaches were clearly limited as evidenced by the fact that they failed to identify all previously known editing sites. What these approaches did reveal though is that recoding sites are just the tip of the iceberg and that the majority of editing occurs in non-coding regions, which are vastly enriched in Alu repetitive elements (Athanasiadis et al., 2004; Blow et al., 2004; Kim et al., 2004; Levanon et al., 2004). These findings explained the abundance of inosine in brain mRNA (Paul and Bass, 1998) and confirmed experimental findings of editing in non-coding regions (Morse et al., 2002). Moreover, since Alu elements are primate-specific and account for >10% of the human genome, A-to-I substitutions are significantly more abundant in primates (Eisenberg et al., 2005). The specific role of these non-coding edits on nervous system operation has not been elucidated. The high abundance of Alus, particularly in gene-enriched regions, will increase the probability for oppositely oriented Alus to anneal into dsRNA secondary structures thus serving as substrates for editing. Alterations of the stability of edited dsRNA structures will affect global RNA metabolism via a link with RNA interference (e.g., Bass, 2006). The advent of high-throughput sequencing technology has led to further advances in our understanding of editing at the genome level and facilitated verification of candidate sites (Li et al., 2009; Wahlstedt et al., 2009), has revealed the interdependence or coupling of multiple editing sites within a transcript (Ensterö et al., 2009), clarified the sequence and structural determinants for editing (Bahn et al., 2012) and enabled a comparison of the sites and frequency of edits between genomes (Danecek et al., 2012). The ongoing efforts of consortia like ENCODE and the 1000 Genomes Project will undoubtedly advance these fronts further (Djebali et al., 2012; Park et al., 2012).

## Outlook

As the discovery of new editing sites continues, so does the need to understand their function, and regulation, in maintaining normal neurophysiology and in mediating adaptability during neuronal plasticity. It is increasingly apparent that the impact of ADARs is widespread, diverse, and under dynamic control, thus the need to dissect the functions of individual editing sites is apparent. Animal models are going some way to achieve this and their contribution to our current understanding have been reviewed (e.g., Rula and Emeson, 2007 and references therein). Recently, new manipulations have emerged that could improve the throughput for investigating the functions of A-to-I editing events, such as the use of substrate-specific helix-threading peptides (Schirle et al., 2010) and steric antisense oligonucleotides (Mizrahi et al., 2013; Penn et al., 2013). Recent advances in the delivery of oligonucleotides using cell-penetrating peptides brings researchers closer to applying these manipulations *in vivo* more routinely (Järver et al., 2012; Moulton, 2012). In the future, these tools together with the increasing capacity of high-throughput resources might lead to therapeutic approaches that could correct defective editing associated with neurological diseases in humans.

### Conflict of interest statement

The authors declare that the research was conducted in the absence of any commercial or financial relationships that could be construed as a potential conflict of interest.
